# Elucidating the cellular actions of demineralised dentine matrix extract on a clonal dental pulp stem cell population in orchestrating dental tissue repair

**DOI:** 10.1177/2041731415586318

**Published:** 2015-05-14

**Authors:** Chi P Lee, John S Colombo, Wayne Nishio Ayre, Alastair J Sloan, Rachel J Waddington

**Affiliations:** 1 Tissue Engineering and Reparative Dentistry, School of Dentistry, Cardiff University, Cardiff, UK; 2 Department of Medicine, Imperial College London, London, UK; 3 School of Dentistry, The University of Utah, Salt Lake City, UT, USA; 4 Cardiff Institute of Tissue Engineering and Repair, Cardiff University, Cardiff, UK

**Keywords:** Dental pulp, mesenchymal stem cells, dentine matrix, cell proliferation, anti-apoptotic, osteogenesis, odontogenesis, dentine repair

## Abstract

Bioactive growth factors identified within the extracellular matrix of dentine have been proposed roles in regulating the naturally inherent regenerative dentine formation seen in teeth in response to trauma and infection, which may also be harnessed for novel clinical treatments in augmenting mineralised tissue repair. This study examined the specific biological action of demineralised dentine matrix extract on a clonal population of dental pulp stem cells in stimulating the prerequisite stages of wound healing associated with mineralised tissue repair. A clonal dental pulp stem cell population with sustained proliferative capacity and multi-potentiality towards osteogenic, adipogenic and chondrogenic lineages was isolated from the pulp of human third molars. Dentine was collected from human healthy teeth, powdered and treated with ethylenediaminetetraacetic acid to obtain a solubilised DDM protein extract. The influence of DDM on the DPSC clonal population was assessed in vitro. Exposure of cells to proteolytically degraded DDM or unsupplemented media served as controls. Compared to controls, DDM stimulated cell expansion, reduced apoptotic marker caspase 3, increased cell survival marker Akt1 and enhanced mineralised matrix deposition as determined by mineral deposition and increased expression of bone-related markers, alkaline phosphatase and osteopontin. Dental pulp stem cells successfully migrated into collagen gels supplemented with demineralised dentine matrix, with cells remaining viable and expanding in numbers over a 3-day period. Collectively, the results provide evidence that soluble proteins extracted from dentine matrix are able to exert a direct biological effect on dental pulp stem cells in promoting mineralised tissue repair mechanisms.

## Introduction

In response to severe trauma or infectious injury, the dentine–pulp complex possesses a natural regenerative ability leading to the rapid deposition of a mineralised matrix at the dentine–pulp interface, immediately below the site of injury, with a primary function to protect the dental pulp from the effects of further insult. This reparative dentine histologically represents an amorphous tissue, with some resemblance of osseous tissue and hence is often also termed ‘osteodentine’. The key biological principles underpinning the reparative process essentially follows a wound repair process involving the recruitment and proliferation of dental pulp stem or progenitor cells (DPSCs) and their subsequent differentiation to what are regarded as odontoblast-like cells which synthesise the mineralised tissue.^1^ The process is complex and still not fully understood in terms of the molecular signals, but a range of growth factors and other bioactive molecules releasable from dentine matrix have been suggested as important contributors in stimulating repair. Harnessing this natural repair process offers a novel potential for demineralised dentine matrix (DDM) to be utilised therapeutically to enhance dentine regeneration to improve longevity of dental tooth restorations and for bone augmentation applications.

Recent proteomic analyses of dentine tissue samples have identified between 179 and 289 different protein components.^2–4^ This has included the definitive identification of transforming growth factor beta 1 (TGF-β1) as a predominant growth factor. The importance of TGF-β1 in the regenerative process has been indicated in previous work where crude TGF-β1-based alginate hydrogels were found to induce de novo dentinogenesis on a cut pulp tissue surface.^5^ Studies have also shown the capacity of bone morphogenetic proteins (BMPs), BMP-2, BMP-4^6^ and BMP-7^7^ to up-regulate dentine matrix synthesis and secretion. However, when used in single growth factor therapy, supraphysiological quantities of proteins are required to illicit biological responses. It is now well recognised that a ‘cocktail’ of growth factors acting synergistically and at nanogram levels is responsible for co-ordinating the reparative events in vivo. Also identified in dentine, are growth factors, such as fibroblast growth factor (FGF), FGF-2, FGF-4 or FGF-10, insulin-like growth factor (IGF) and vascular endothelial growth factor (VEGF), which have collectively been implicated in the recruitment and differentiation of mesenchymal stem cells (MSCs) towards an odontoblast or osteoblast lineage, in addition to stimulating endothelial cells angiogenesis.^5,8–11^ The identification within the dentine matrix of pro-inflammatory cytokines, including interleukin (IL)-2, IL-6 and IL-8, and anti-inflammatory cytokines, IL-4 and IL-10,^12^ supports proposals that DDM may also have a role in supporting the inflammatory process necessary for initiating tissue regeneration. The dentine matrix is also well characterised with respect to non-collagenous components, which include the small leucine-rich proteoglycans (SLRPs), decorin and biglycan, which have been proposed to sequester and protect the growth factors from proteolysis and have purported roles for indirectly modulating cell signalling.^13–18^ Equally, matrix proteins such as dentine sialoprotein, dentine phosphoprotein, bone sialoprotein (BSP), osteopontin (OPN), dentine matrix protein-1 (DMP-1) and matrix extracellular phosphoglycoprotein (MEPE) are all identifiable within the matrix with various proposed signalling roles in the early reparative events influencing cell survival, cell differentiation and regulating mineral deposition.^19^ The dentine matrix also contains matrix metaloproteinases (MMP-2, -8, -9, -13 and -20) which have been suggested to enhance bioactivity through proteolytic activation of growth factors and signalling molecules such as DMP-1.^19^ MMP-2 and MMP-3 have been reported to form complexes with BSP and OPN, respectively,^20^ enhancing proteolytic activity and degradation products of the extracellular matrix (ECM) may partake in stimulating immune responses which subsequently initiate wound repair.^21^

While proteomic studies demonstrate a bioactive potential for DDM, only a handful of studies have demonstrated its ability to stimulate DPSC populations to synthesise a mineralised matrix. In vitro, lyophilised DDM has been shown to mediate dental papilla cell differentiation,^22^ and soluble extracts from whole teeth have been shown to stimulate mineralised matrix formation by heterogeneous populations of DPSC.^3^ Similarly, in vivo studies have demonstrated increased dentine secretions around dentine chips placed in the pulp chamber^23^ and by soluble tooth extracts placed in rat tooth socket^24^ or incorporated into embryonic tooth buds transplanted under the renal capsule of mice.^3^ Similar studies have also demonstrated its potential to considerably stimulate bone repair in vivo in a rabbit critical size defect.^25^ However, it is now well recognised that the resident DPSC population is heterogeneous in nature. Studies on clonal populations expanded from single cells have indicated populations within two extremes: cells with high colony-forming efficiency, long-term proliferative capacity and multi-potency for lineage differentiation, including osteogenesis, chondrogenesis and adipogenesis, and cells with limited proliferative lifespan and restrictive differentiation for a specific lineage such as osteogenesis.^26–28^ The various populations are proposed to yield different responses in wound repair process, although exact contributions remain undefined. Significantly, diversity in the heterogeneous profile is predicted between donor tissue sources which have the potential to impact in direct comparison of results and in identifying a clear assessment of biological behaviour. Against this background, the aim of this study is to investigate the effect of a DDM protein extract derived from human teeth on a clonal population of DPSCs. To provide greater competency in the results, a clonal population, characterised by substantial immature or embryonic characteristics, high proliferative capacity and multi-potency, was selected for use in the study. This provided the baseline characteristics of a cell population for better assessment of biological roles for DDM in stimulating cell migration, expansion, survival and driving osteoblastic differentiation, as prerequisites for the dentine repair process.

## Methods and materials

### Preparation of DDM

DDM was prepared from extracted healthy human teeth obtained from the Oral Surgery Department at the Birmingham Dental Hospital, collected along with patient informed consent for preparation of DDM prior to 2004 Human Tissue Act, United Kingdom. Teeth were sectioned down the buccal–lingual plane; pulpal tissue was extirpated; and cementum, enamel and carious tissue were removed with a slow-speed dental hand piece with a diamond-tipped burr. Remnant dentine was powdered using a ball mill in liquid nitrogen and then treated with 10% ethylenediaminetetraacetic acid (EDTA) (pH 7.2), containing protease inhibitors, 1 mM iodoacetic acid, 5 mM n-ethylmalemide and 5 mM benzamidine HCl at 4°C for 14 days, to solubilise non-collagenous proteins and soluble collagens as the tissue demineralised. Every 2 days, the EDTA extractant was recovered by centrifugation at 1000g for 10 min, and the residual dentine matrix was retreated with further EDTA. All protein extracts were pooled, and the EDTA and protease inhibitors were removed by exhaustive dialysis against double distilled water, and the DDM was recovered by lyophilisation. For a sample of DDM, non-collagenous and collagenous proteins were removed or denatured to provide a negative control (DDM-neg). A total of 100 µg DDM powder was denatured with 100 µL 4 mg/mL collagenase or dispase (Roche Applied Science, United Kingdom) at 37°C for 1 h. The DDM-neg was exhaustively dialysed against double distilled water at 4°C and lyophilised. DDM-neg was reconstituted in 1 mL of basal alpha Minimum Essential Medium (α-MEM) to produce a concentration equivalent to 100 µg/mL of DDM before denaturation.

### Isolation of DPSC clones

Human third molars (18–35 years old) were obtained from patients attending the School of Dentistry, University Hospital of Wales, Cardiff, United Kingdom. Local ethical committee approval was obtained for this study from the South East Wales Research Ethics Committee of the National Research Ethics Service (NRES), UK Ref 12/WA/0107, approving the process for obtaining and recording written consent within the School of Dentistry, Cardiff, United Kingdom. Outer surfaces were sterilised with industrial methylated spirits (IMS) for 10 s. Teeth were split laterally, and exposed pulps were immediately extracted and incubated with 4 mg/mL collagenase or dispase (Roche Applied Science) for 1 h at 37°C. Enzymic activity was stopped by the addition of 5–10 mL basal α-MEM (supplemented with ribonucleosides and deoxyribonucleosides; Gibco, Invitrogen, United Kingdom); 20% heat-inactivated foetal calf serum (hi-FCS; Invitrogen); 4 mM l-glutamine (Gibco); 100 µM l-ascorbate 2-phosphate (Sigma–Aldrich, United Kingdom); and 100 units/mL penicillin G sodium, 0.1 µg/mL streptomycin sulphate and 0.25 µg/mL amphotericin (Gibco). Cells were passed through a 70-µm cell strainer (BD Falcon, United Kingdom) and then twice pelleted by centrifugation at 400g, for 5 min and resuspended in the serum-free basal α-MEM to ensure removal of enzyme and serum.

Immature progenitor cells expressing high surface levels of functional β1 integrin were selected by differential fibronectin adhesion as previously described by Waddington et al.^29^ and Dowthwaite et al.^30^ Briefly, cells in serum-free media, were seeded onto fibronectin-coated six-well plates at 4 × 10^3^ cells/cm^2^ for 20 min at 37°C. Non-adherent cells were removed, and adherent cells were allowed to form single-cell colonies by continued culture in the serum supplemented basal α-MEM at 5% CO_2_ and 37°C. Colonies of >32 cells were dissociated with 50 µL trypsin enclosed within the cloning ring and seeded into a 96-well plate. Cells were passaged on reaching 80%–90% confluency, maintaining a reseeding density of 5 × 10^3^/cm^2^, and culture expanded into larger T75 flasks. Population doublings (PDs) were calculated from cell counts at each passage using the formula PD = (log10 (total cell count obtained) − log10 (total cell count re-seeded/log10 (2)). Analyses described below were performed at 21–24 PDs.

### Reverse transcriptase–polymerase chain reaction

Reverse transcriptase–polymerase chain reaction (RT-PCR) was used to indicate gene expression of mesenchymal and embryonic stem cell markers and osteogenic, adipogenic and chrondrocytic differentiation makers. Total cellular RNA was extracted from cells using a Qiagen RNeasy Mini kit (Qiagen Ltd, United Kingdom) according to manufacturer’s protocol, and quantity and purity of RNA was determined by absorbance at 260 or 280 nm. Complementary DNA (cDNA) was synthesised using 1 µL Moloney murine leukaemia virus (M-MLV) reverse transcriptase, 0.625 µL of RNasin, 1.25 µL of 10 mM deoxynucleotide triphosphates (dNTPs; all Promega, United Kingdom) at 25°C for 10 min, 37°C for 1 h, followed by 95°C for 5 min. Polymerase chain reactions (PCRs) were performed using GoTaq Flexi DNA polymerase, dNTPs, relevant reverse and forward primer sequences (as detailed in Table 1) and the following cycling conditions: initial denaturation at 94°C for 5 min; 40 cycles of denaturing, annealing and extension at 94°C for 1 min, 62°C for 30 s and 72°C for 1 min, respectively; and final extension at 72°C for 10 min. RT-PCR products were separated by electrophoresis on 1.5% agarose gels containing ethidium bromide and viewed under ultraviolet (UV) light. β-Actin was used as a housekeeping gene for RT-PCR.

**Table 1.** Forward and reverse primer sequences used in RT-PCR reactions.

RT-PCR primer	Sequence (5’–3’)	Anneal temperature (°C)	Product size (bp)
CD105	FWD: GAAGGGCTGCGTGGCTCAGG	55	483
	RVS: CCTTCCAAGTGGCAGCCCCG		
CD146	FWD: CGACAACGGGGTCCTGGTGC	55	321
	RVS: CAGCGATAGCCGCCTCCTGC		
MSX-1	FWD: GAAGCCCGAGAGGACCCCGT	55	410
	RVS: AGGCACCGTAGAGCGAGGCA		
P75/CD271	FWD: CTGCAAGCAGAACAAGCAAG	55	310
	RVS: GGCCTCATGGGTAAAGGAGT		
Slug	FWD: GAGCATACAGCCCCATCACT	55	479
	RVS: CTCCCCCGTGTGAGTTCTAA		
Snai1	FWD: GCGAGCTGCAGGACTCTAAT	55	441
	RVS: CCAGGCTGAGGTATTCCTTG		
Twist 1	FWD: GTCCGCAGTCTTACGAGGAG	55	711
	RVS: GATGCCTTTCCTTTCAGTGG		
hTERT	FWD: CGGAAGAGTGTCTGGAGCAA	55	145
	RVS: GGATGAAGCGGAGTCTGGA		
hTR	FWD: CTAACCCTAACTGAGAAGGGCGTA	55	154
	RVS: GGCGAACGGGCCAGCAGCTGACATT		
Oct4(a)	FWD: AGGAGTCGGGGTGGAGAG	55	250
	RVS: CGTTTGGCTGAATACCTTCC		
Nanog	FWD: CAAAGGCAAACAACCCACTT	55	432
	RVS: CAGGACTGGATGTTCTGGGT		
Notch 1	FWD: CTACCTGTCAGACGTGGCCT	55	356
	RVS: CGCAGAGGGTTGTATTGGTTCG		
Notch 2	FWD: AAGCAGAGTCCCAGTGCCTA	55	172
	RVS: CAGGGGGCACTGACAGTAAT		
Notch 3	FWD: CAGTCGCCTGAGAATGATCAC	55	195
	RVS: GAATGACCAGCAGCAAGACAG		
Jagged 1	FWD: GACTCATCAGCCGTGTCTCA	55	190
	RVS: CTGGGGAACACTCACACTCAA		
Jagged 2	FWD: CTACAATGGTGGCATCTGTG	52	156
	RVS: GCGATACCCGTTGATCTCAT		
Osteocalcin	FWD: GCAGGTGCGAAGCCCAGCGGTGCAGAG	62	341
	RVS: GGGCTGGGAGGTCAGGGCAAGGGCAAG		
Bone sialoprotein	FWD: GGGCTATGGAGAGGACGCCACGCCTGG	62	340
	RVS: CGAGGTGCCCTTGCCCTGCCTTCCGGTC		
Sox9	FWD: GTGAACTGGCCACCCCGCGCCTTCCTA	62	936
	RVS: CAGCCTTGCCCGGCTGCACGTCGGTTT		
Type 2 collagen	FWD: GGCTGGCAGCTGTGTGCAGGATGGGCA	62	930
	RVS: GCGCCAGCAGGGCCAGTCCGTCCTCTT		
Lipoprotein lipase (LPL)	FWD: GCTGGCATTGCAGGAAGTCTGACCAATAAG	56	621
	RVS: GGCCACGGTGCCATACAGAGAAATCTCAAA		
PPAR-γ2	FWD: GCCATCAGGTTTGGGCGGATGCCACAG	62	349
	RVS: CCTGCACAGCCTCCACGGAGCGAAACT		
Caspase 3	FWD: GTTTGTGTGCTTCTGAGCCA	55	350
	RVS: TCAAGCTTGTCGGCATACTG		
Akt1	FWD: GTGCCACCATGAAGACCTTT	55	459
	RVS: CATCTTGGTCAGGTGGTGTG		
β-Actin	FWD: AGGGCAGTGATCTCCTTCTGCATCCT	65	480
	RVS: CCACACTGTGCCCATCTACGAGGGGT		

RT-PCR: reverse transcriptase–polymerase chain reaction; FWD: forward; RVS: reverse.

β-Actin was used as a housekeeping gene.

### Adipogenic differentiation of DPSC clonal cells

Cells, at 24 PDs and 80%–90% confluency, were cultured in 5% CO_2_ at 37°C following the sequence 72 h in α-MEM, 10% hi-FCS, 10 µg/mL insulin, 1 µM dexamethasone, 100 µM indomethacin and 100 µM 3-isobutyl-1-methylxanthine (IBMX) with antibiotics or antimycotics (adipogenic induction media) followed by 24 h in α-MEM, 10% hi-FCS, 10 µg/mL insulin and antibiotics or antimycotics (adipogenic maintenance media) for four rotations (total: 16 days) before culturing cells in adipogenic maintenance media for a final 7 days.^23^ Cells cultured in the basal α-MEM were used as a negative control. Cells were stained with Oil Red O and viewed under a light microscope. Cultures were also prepared to examine messenger RNA (mRNA) expression levels for lipoprotein lipase (LPL) and peroxisome proliferator-activated receptor gamma-2 (PPAR-γ2) by RT-PCR.

### Chondrogenic differentiation of DPSC clonal cells

A total of 2.5 × 10^5^ cells (at 24 PDs) were pelleted in a 15-mL polypropylene conical base tubes (Falcon, United Kingdom) by centrifugation at 150g for 5 min and then cultured in 1 mL non-haematopoietic (NH) stem cell media ChondroDiff medium (Miltenyi Biotec, United Kingdom) at 5% CO_2_ and 37°C. As a negative control, cells were cultured in the basal α-MEM media. After 24 days, pellet cultures were fixed overnight in neutral-buffered formalin, dehydrated by passing through ascending alcohol concentrations and paraffin wax embedded. A total of 5-µm sections were prepared on poly-l-lysine–coated glass slides. Sections were deparaffinised with xylene and rehydrated and treated with 0.15 U/mL chondroitinase ACII (SeikaGaku Kogyo Co., Japan), Tris acetate, pH 8, for 30 min at 37°C to reveal digested neo-epitopes. Sections were blocked with 1% bovine serum albumin (BSA) and Tris-buffered saline (TBS) for 30 min and then incubated with monoclonal immunoglobulin G (IgG) antibody 2-B-6 (detects chondroitinase neo-epitopes; dilution 1:500; gift from B. Caterson, Cardiff University, United Kingdom) for 1 h. Immuno-reactivity was detected with goat anti-mouse IgG-fluorescein isothiocyanate (FITC; dilution 1:200; Santa Cruz Biotechnology, Inc, Texas, USA) for 45 min. Nuclear material was stained with bisbenzimide. Cell cultures were also established to examine mRNA expression for Sox9 and type 2 collagen by RT-PCR.

### Osteogenic differentiation of DPSC clonal cells

Cells at 24 PDs were seeded at 2200 cells/cm^2^ on round glass slides in six-well plates and cultured for 23 days in the above basal α-MEM supplemented with 10 nM dexamethasone and 100 µM β-glycerolphosphate (both from Sigma–Aldrich, United Kingdom) at 5% CO_2_ and 37°C. Cells cultured in the absence of dexamethasone and β-glycerolphosphate served as a negative control. For some cultures (n = 3), cells were stained with 20 g/L Alizarin Red, pH 4.2 for 2–5 min. Cultures were also prepared to examine mRNA levels for osteocalcin (OCN) and BSP by RT-PCR.

### Influence of DDM on cell expansion

Clonal DPSCs (21–24 PDs) were seeded into 96-well plates at 1.5 × 10^4^ cells/cm^2^ and cultured in 200 µL basal α-MEM overnight at 37°C and 5% CO_2_. Media was then replaced with 200 µL basal α-MEM supplemented with 0–10 µg/mL DDM or 0–10 µg/mL DDM-neg (concentrations equivalent to the DDM before denaturation) served as a negative control. Media was refreshed every 24 h. At 24, 48 and 72 h, cells were fixed overnight at 4°C in 70% ethanol and then stained with 0.1% crystal violet (BDH Laboratory Supplies, United Kingdom), 0.1 M borax, 2% ethanol, pH 9 for 1 h with gentle agitation, rinsed well with water and counted when viewed under a light microscope. Counts were obtained from triplicate wells, and the assay was repeated on three separate occasions.

### Influence of DDM on cell apoptosis

Clonal DPSCs at 20–24 PDs were seeded into 96-well plates at 1.5 × 10^4^ cells/cm^2^ and cultured in 200 µL of the DDM, DDM-neg supplemented or unsupplemented α-MEM as described above. After 24, 48 and 72 h, apoptosis was measured using the Caspase-Glo 3/7 Assay (Promega, United Kingdom) following the manufacturer’s protocol. The assay was performed in triplicate on three separate occasions. Cell cultures were also established to examine mRNA expression for caspase 3, Akt1 and proliferating cell nuclear antigen (PCNA) by RT-PCR.

### Influence of DDM on DPSCs migrating into collagen gels

Collagen gels (1 mg/mL final concentration) were prepared by mixing 1.79 mL liquid rat tail collagen I (supplied as 3.91 mg/mL in 0.02 N acetic acid; BD Biosciences, United Kingdom) with 5.17 mL of the above serum supplemented α-MEM. Gels contained either DMP or DMP-neg 0–10 µg/mL (final concentration). pH was neutralised with 41 µL of 1 N NaOH, and 1 mL gel mixture was immediately transferred into 12-well tissue culture plates to solidify overnight. A total of 400 µL of basal α-MEM was layered onto the surface of each gel, and tissue culture inserts with 8-µm pore membranes (Greiner Bio-One Ltd, Glasgow, UK) were placed into the wells in contact with the collagen gels. DPSCs at 20–24 PDs were immediately seeded into the well insert at 1 × 10^4^/cm^2^ in unsupplemented α-MEM and cultured at 37°C and 5% CO_2_. After 24 and 72 h, gels were fixed with 4% paraformaldehyde for 30 min and then permeated with 0.3% triton-X 100 for 30 min. Gels were washed with TBS prior to staining with phalloidin-FITC (Sigma–Aldrich) in 1% BSA or TBS at 4°C for 1 h. Cell nuclear material was stained with bisbenzimide (Sigma–Aldrich) for 30 min at 4°C and then washed gels were viewed by fluorescence microscopy. Bisbenzimide-stained nuclei were automatically counted using ImageJ software to obtain cell counts. Cell counts were obtained from five random fields of view (10× magnification) from triplicate wells for each experimental condition. Depth of migration was assessed from Z-stacks of gels viewed by confocal microscopy.

### Influence of DDM on osteoblast differentiation

DPSCs at 20–24 PDs were seeded into six-well plates at 2200 cells/cm^2^ and cultured in 0 or 10 µg/mL DDM, supplemented or unsupplemented α-MEM as described in the above section. After 3, 10 and 21 days, total mRNA was isolated from the cells, and cDNA was synthesised as described above. mRNA levels for OPN and alkaline phosphatase was quantified by quantitative PCR (qPCR) using SYBR Green JumpStart Taq readymix following the manufacturer’s protocol, using the forward and reverse primers as detailed in Table 2. For all qPCR analyses, glyceraldehyde-3-phosphate dehydrogenase (GAPDH) was used as the housekeeping gene for relative quantification. In a separate experiment, cells were cultured for 5 and 20 days in the presence and absence of 10 µg/mL DDM and stained with Alizarin Red for 5 min.

**Table 2.** Forward and reverse primer sequences used in qPCR reactions.

qPCR primers	Sequence (5’–3’)	Annealing temperature (°C)	Product size (bp)
Osteopontin	FWD: ATCACCTGTGCCATACCA	55	430
	RVS: CATCTTCATCATCCATATCATCCA		
Alkaline phosphatase	FWD: GGACCATTCCCACGTCTTCAC	55	137
	RVS: CCTTGTAGCCAGGCCCATTG		
GAPDH	FWD: GGTCGGAGTCAACGGATT	55	253
	RVS: ATCGCCCCACTTGATTTTG		

GAPDH: glyceraldehyde-3-phosphate dehydrogenase; qPCR: quantitative PCR; FWD: forward; RVS: reverse.

GAPDH was used as a housekeeping gene.

### Statistical analysis

Where applicable, average values and standard error of mean (SEM represented as ±) were determined using statistical software GraphPad Instat. In order to analyse differences between the various supplementation conditions, values obtained were compared using analysis of variance (ANOVA) with a post hoc Tukey–Kramer multiple comparisons test. p-values of <0.05 were considered statistically significant at a 95% confidence interval.

## Results

### Characterisation of clonal DPSC population

**Figure 1.**
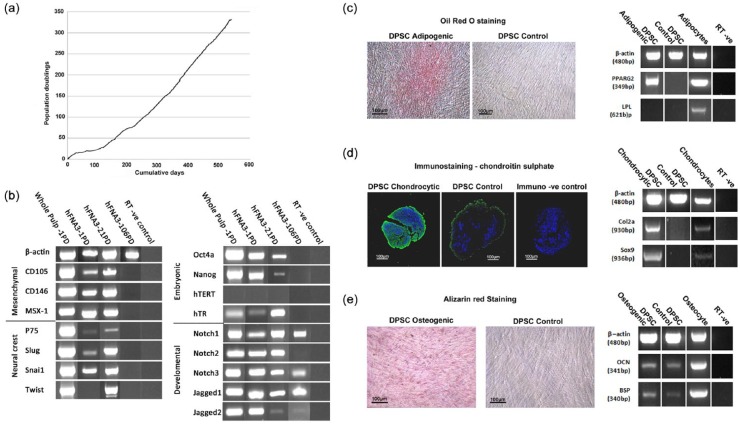
Characterisation of the selected clonal cell population isolated from dental pulp demonstrating the isolation of an immature mesenchymal progenitor population with long proliferative lifespan and multi-potency: (a) isolated clone, designate hFNA3 achieved over 325 PDs following culture expansion; (b) RT-PCR analysis demonstrated the presence of mRNA expression for mesenchymal, neural crest, embryonic and developmental cell markers during early population doublings, which were lost at 106 PDs. Also shown is the presence of the markers in cells derived from whole dental pulp, representing a positive control; (c) demonstration for the expression of adipogenesis by Oil Red O staining and mRNA expression of early marker proliferator-activated receptor gamma (PPAR-γ) but not the later expressing marker lipoprotein lipase (LPL); (d) chondrogenesis by immunostaining for chondroitin sulphate proteoglycans and mRNA expression of type 2 collagen (Col2a) and SOX9; and (e) osteogenesis by Alizarin Red staining for mineral deposits and mRNA expression for osteocalcin (OCN) and bone sialoprotein (BSP). For all images, scale bar represents 100 µm.

Immature mesenchymal progenitor cells were selected by preferential adhesion to fibronectin over a 20-min incubation time. Low-density seeding allowed clonal colonies to develop over a 12-day culture period. Isolated clonal colonies were culture expanded and one clone, designate hFNA3, was selected for this investigation following the definitive identification of MSC characteristics (Figure 1). This particular clone demonstrated an extensive proliferative lifespan beyond 325 PDs (Figure 1(a)). Following establishment of sufficient cell numbers of the clone in culture and at 21 PDs, hFNA3 expressed a triplicate of mesenchymal markers, CD105, CD146 and MSX-1 and neural crest markers of p75, slug, snai1 and twist (Figure 1(b)). The embryonic nature of the isolated clone was further demonstrated by expression of Oct4a and Nanog. The presence of these mesenchymal, neural crest and embryonic markers in whole pulp is shown as a positive control, demonstrating the presence of a similar clonal population within the heterogeneous MSC populations of the original pulpal tissue. Telomerase reverse transcriptase in humans (hTERT) was not detected in either the clonal population or the whole pulpal tissue, although its RNA encoding domain hTR was expressed. Early developmental markers, notch receptors Notch 1, 2 and 3, with associated ligands Jagged 1 and 2 were also expressed. Cells following 106 PDs failed to express any of the stem cell markers analysed but did retain expression for Notch 1 and 3 and Jagged 1 and 2 (Figure 1(b)). Based on these results, multi-potency was assessed for hFNA3 clonal cells at 24 PDs (Figure 1(c)–(e)). Culture of DPSCs in adipocytic media for a total of 23 days resulted in the induction of PPAR-γ2 mRNA (Figure 1(c)). LPL mRNA was not detected, while low levels were detected in the adipocyte positive control, possibly indicating that the DPSCs had not achieved full differentiation. However, adipocytes exhibited Oil Red O–positive lipid-filled vacuoles. Following culture of DPSCs in chondrocytic media for 24 days, strong expression for type 2 collagen and Sox 9 mRNA was apparent (Figure 1(d)). Immunocytochemistry indicated high staining for chondroitin sulphate around cells indicative of high levels of proteoglycan synthesis (Figure 1(d)). Following culture in osteogenic media for 23 days, an osteoblast phenotype was identified as determined by mRNA expression of OCN and BSP, and the initial formation of mineralising foci which stained with Alizarin Red (Figure 1(e)).

### Influence of DDM on DPSC cell viability and apoptosis

**Figure 2.**
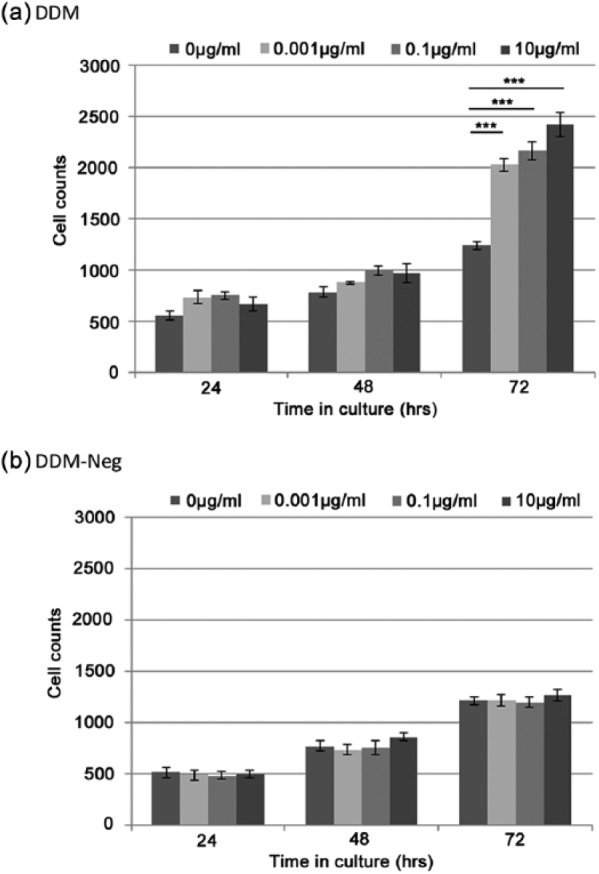
Stimulatory effect of DDM on the expansion of hFNA3 clonal cell numbers during culture over 72 h: (a) cells were visualised by staining with crystal violet, and average cell counts and corresponding SEMs were obtained (n = 9). As a negative control, DDM was denatured to produce DDM-neg, where bioactivity was observed to be abolished, and (b) concentrations reported for DDM-neg are equivalent to the starting concentration of DDM prior to denaturation treatment. *p < 0.05, **p < 0.01 and ***p < 0.001.

The addition of DDM to the culture media induced a significant increase in the number of viable cells (Figure 2(a)), with all concentrations analysed demonstrating significant differences in cell numbers at 72 h compared with the unsupplemented control (p < 0.001). The effect of DDM on cell expansion was dose dependent. As a negative control, supplementation with DDM-neg, where the protein matrix had been proteolytically degraded, indicated that stimulatory activity of the DDM had been abolished. At all concentrations examined (equivalent to the starting concentration of the DDM), there was no difference in the expansion of viable cell numbers compared with the unsupplemented control (Figure 2(b)).

**Figure 3.**
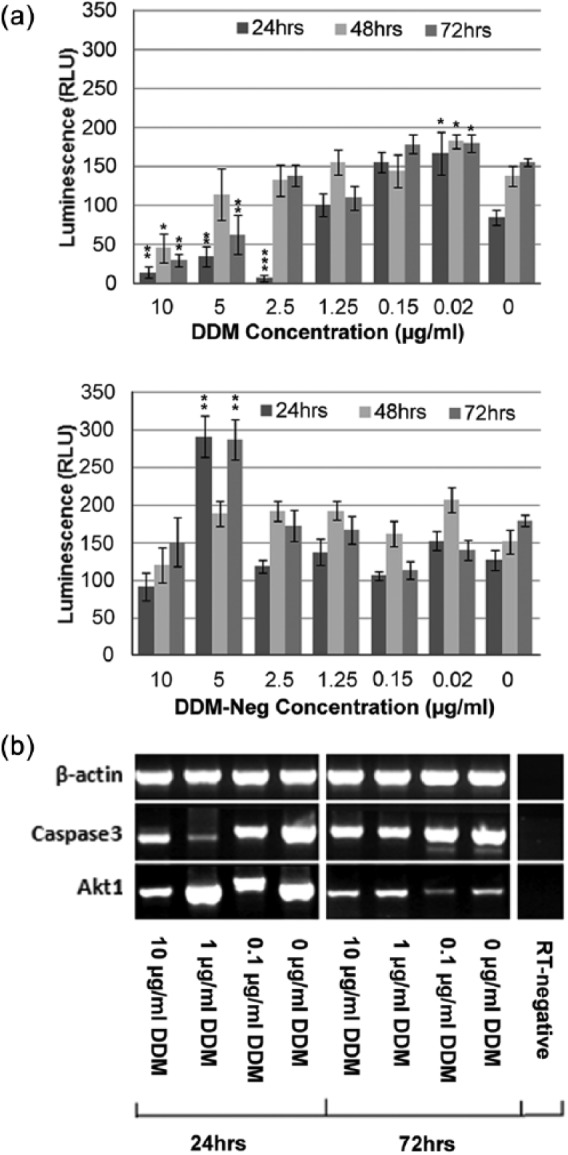
(a) Anti-apoptotic effect of DDM on the DPSC clonal cells as demonstrated by reduced caspase 3 activity. A similar effect was not observed following supplementation with DDM-neg, and apoptosis was stimulated in the presence of the degraded products at 5 µg/mL. (b) RT-PCR for mRNA expression levels of caspase 3 and the cell survival factor Akt1 confirm the results of the caspase assay. Means and SEMs for the caspase 3 luminescence assay are calculated from n = 9. *p < 0.05, **p < 0.01 and ***p < 0.001.

The culture of DPSC in the presence of greater than 2.5 µg/mL DDM significantly reduced apoptotic caspase 3 activity compared to the unsupplemented (0 µg/mL) control cultures (Figure 3(a)). This effect was not apparent when cells were supplemented with DDM-neg at these concentrations; indeed at 5 µg/mL, apoptotic caspase 3 was significantly increased compared to the unsupplemented culture (p < 0.001). At concentrations 1.25–0.08 µg/mL DDM, there was no significant difference on the level of apoptotic caspase 3 compared to unsupplemented control. Conversely, at lower concentrations of 0.04–0.01 µg/mL, there appeared to be an increase in caspase 3 activity compared to levels at similar time points for 0 µg/mL. These data are partially supported by the analysis of mRNA which also indicated a substantial reduction in caspase 3 mRNA and considerable increase in cell survival protein Akt1 in the presence of 1 µg/mL DDM at 24 h (Figure 3(b)). Expression levels resumed to basal unsupplemented levels by 72 h.

### Influence of DDM on DPSCs migrating into collagen gels

**Figure 4.**
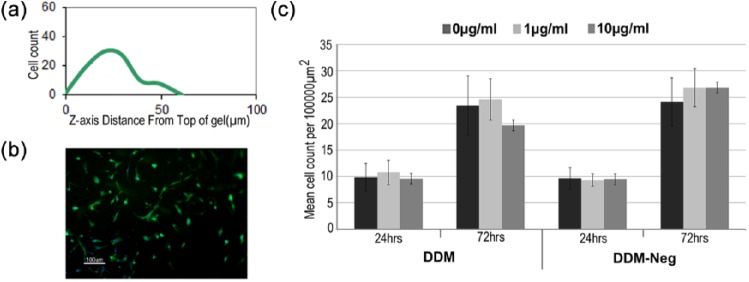
DDM has no stimulatory or impeding influence on the migration of DPSC clonal cells into collagen gels: (a) typical confocal data derived from Z-stacks obtained from confocal microscopy images of gels indicating the ability of the cells to migrate into the collagen matrix to a depth of 50 µm; (b) collagen gels were also examined by fluorescence microscopy, with cells viewed from the top surface and cell count, using ImageJ, and are shown graphically for the respective experimental and control groups after 24 and 72 h in culture; and (c) means and SEMs are calculated from n = 15; statistical analysis indicated no significant difference between gels.

Confocal microscopy of phalloidin-FITC-stained cells indicated that DPSCs were able to migrate into the collagen gels to a depth of approximately 50 µm, regardless of whether the gels contained DMP, DMP-neg or were unsupplemented (Figure 4(a)). For all gels, the cells appeared to be evenly distributed throughout (Figure 4(b)). Average cell counts and standard deviations were obtained from five images obtained from three separate gels. The results obtained indicate that supplementation with either DDM or DDM-neg does not enhance or impede cell migration through the surface layers of the collagen gels (Figure 4(c)). After 24 h, the presence of neither 1 nor 10 µg/mL DDM did not significantly increase the number of cells entering the gel compared with the unsupplemented control (p > 0.05). Similar results were obtained for DDM-neg (p > 0.05). At 72 h, an increase in cell counts was observed, possibly through continued migration or proliferation of the cells in the gels, but again, no significant difference was seen due to the supplementation of the gels with DDM or DDM-neg.

### Influence of DDM on osteoblast differentiation

**Figure 5.**
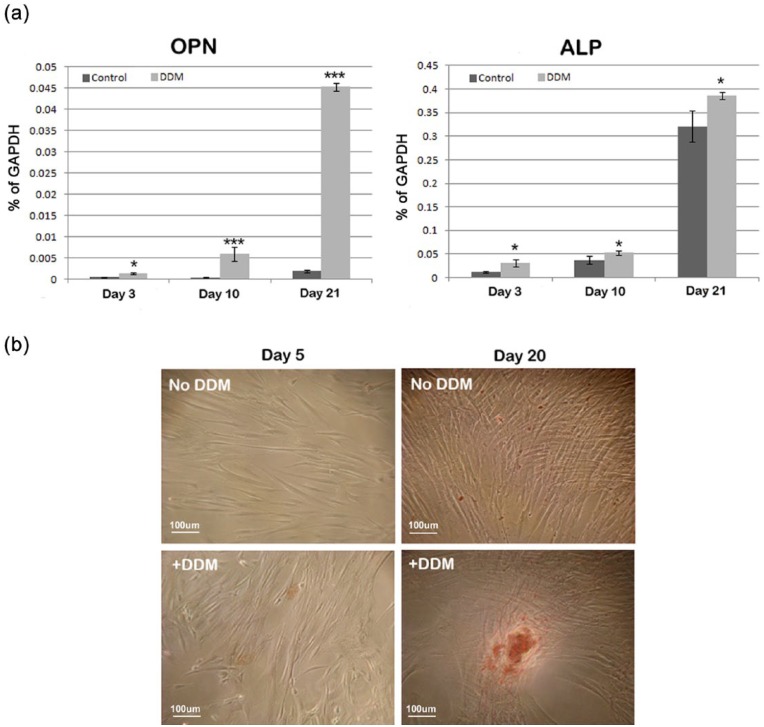
The ability of DDM to induce osteogenesis: (a) mRNA expression of osteogenic markers osteopontin (OPN) and alkaline phosphatase (ALP) as determined by quantitative PCR, and (b) the appearance of Alizarin Red staining mineral deposits and mineralised nodules in the presence and absence of DDM. Means and SEMs are calculated from n = 6. *p < 0.05, **p < 0.01 and ***p < 0.001.

The presence of 1 µg/mL DDM in the culture media stimulated the expression of mRNA levels for OPN and alkaline phosphatase (Figure 5). qPCR analysis indicated significant increases for osteogenic markers, OPN and alkaline phosphatase at all days examined during osteogenic induction (p < 0.05 for alkaline phosphatase at all time points and p < 0.001 for OPN for culture time greater than 10 days). These data are supported by Alizarin Red staining which indicated, in the presence of DDM, the initial formation of mineral deposition following culture for 5 days and the clear formation of mineralised nodules following 20 days in culture.

## Discussion

Protein analysis of DDM suggests that it contains a range of growth factors and matrix proteins which have a bioactive potential for stimulating mineralised tissue repair. While this has been confirmed through in vivo studies, this study is the first to confirm bioactivity on a clonal MSC population derived from human dental pulp. This study specifically identified that DDM has the potential to stimulate cell expansion, reduce apoptosis and induce synthesis of a mineralised matrix, with all responses observed to be concentration dependent. Significantly, these results indicate that physiological levels of bioactive components within the DDM are capable of stimulating the respective cellular events associated with reparative dentine formation allowing further confirmation of in vivo histological studies and proposals derived from the biochemical analysis of the dentine.

In vitro phenotypic data derived from recent studies of clonal populations of MSCs isolated from bone marrow, umbilical cord and dental pulp tissue have led to the proposal that MSCs resident in connective tissue represent a heterogeneous population which can best be presented as a hierarchical structure with respect to differentiation potential and proliferative capacity observed.^27,31–33^ At the head of this structure are the more immature MSCs, most recently derived from the mother stem cell which have a high proliferative capacity and appear multi-potent in nature. As these transit-amplifying (TA) cells rapidly proliferate, they are proposed to become progressively more bi-potent and subsequently unipotent for osteo- (osteodenine-like), adipo- or chondrogenic lineages with limited proliferative capacity.^31–33^ The heterogeneous population in vivo is proposed to contain the complete hierarchical organisation of cells, and donor variability in heterogeneity has the potential to impact on the clarity of the results obtained. Which MSC population contributes directly to reparative dentine formation is not fully clear. Implantation of mouse dental pulp clonal MSC populations into mandibular incisors suggest that those MSCs which demonstrate high multi-potential phenotype in vitro contribute better to reparative dentine formation in vivo compared with the lineage-restricted cells.^34^ In situ injury studies tracking the different MSC phenotypes have, however, suggested that the lineage-restricted TA cells with presumed unipotency for osteogenesis or osteodentine formation, are the first to differentiate into new odontoblast-like cells following cavity-induced injury, with these cells replenished by the more multi-potent cells.^35^ Both studies would indicate an importance for the multi-potent cell, and consequently, this study utilised a clonal cell line of defined immature mesenchymal physiognomies, which demonstrated an extensive proliferative lifespan and had not become lineage restricted. We are, thus, confident that the bioactivity of DDM was assessed on a well-characterised multi-potent cell population which had no preferential differentiation for osteogenic or odontogenic lineages. Expansion of the cells to 106 PDs resulted in the loss of all except for Notch 1 and 3 and Jagged 1 and 2. The change in expression profile indicates the loss of the immature stem cell characteristics (supported by the loss of embryonic and MSC markers), correlating with a loss in multi-potency at later PDs (results not shown). Notch 2 is not expressed in the pulp of healthy adult teeth, but expression is up-regulated during dentine repair, where it is proposed to have a role in enhancing survival of uncommitted precursors and intermediate-stage cells within the sub-odontoblast region.^36^ The assessment of DDM bioactivity was, thus, performed using clonal cells at a low passage (24 PDs) where the regenerative potential of high proliferation and multi-differentiation capacity was optimal.

Dentine regeneration occurs within the mature adult tooth either as a result of caries or trauma resulting in significant loss of the primary dentine (formed during embryonic tooth development) and hence the loss of the embedded mature odontoblasts cells which maintain the vitality of the tissue. The regeneration process involves the migration of MSCs to an area below the site of injury, followed by their cellular expansion and subsequent differentiation to odontoblast-like cells that produce a mineralised ‘barrier’ tissue directly below the devitalised dentine. Unlike the original primary or ‘orthodentine’ which has a defined tubular structure, the synthesised regenerative dentine has the histological appearance of an amorphous mineralised matrix with sporadic cellular inclusions. Biochemically, it shows many similarities to osseous tissue rather than primary dentine, with the prominent presence of bone matrix proteins of BSP and OCN and the notable absence of dentine phosphoprotein.^37^ The molecular regulation of the reparative dentine formation is still not fully established but is reported to involve many of the growth factors that control intramembraneous ossification. Within this study, bioactive factors within the DDM across the concentration range of 0.001–10 µg/mL were shown to stimulate expansion of the clonal DPSCs. The effect was concentration dependent with 10 µg/mL demonstrating the greatest potency. At the higher concentrations of 5–10 µg/mL, the DDM also appeared to aid cell survival through a decrease in the levels of caspase 3 and increase in the expression of the serine threonine kinase, Akt1, which is reported to inactivate apoptotic mechanisms within cells.^38^ Platelet-derived growth factor (PDGF), identified in DDM, has been shown to activate the Akt1 and Akt2 pathways in promoting this anti-apoptotic activity,^39^ but additionally, studies have indicated that the abundant matrix component of dentine phosphoprotein also has an anti-apoptotic activity.^40^ The observed expansion in cell numbers is also likely to be attributable to a range of growth factors with known mitogenic properties for MSCs and bone forming cells, including TGF-β1, PDGF, FGF and IGF-I. Significantly, the synergistic action of these ‘cocktails’ of growth factors collectively facilitates greater signalling power at substantially lower concentration, compared with the studies that have examined the effects of a single growth factor added exogenously. These growth factor ‘cocktails’ are also known to play important active roles during early events of reparative mineral tissue formation.^41^ Within this study, DDM was shown to possess an osteogenic potency, with the deposition of a mineralised matrix and a concomitant increase in OPN and alkaline phosphatase achievable at concentrations of 1 µg/mL. Growth factors present during early healing events are proposed to subsequently stimulate osteogenesis. For example, TGF-β1 has also been proposed to initiate signalling for BMP synthesis by osteoprogenitor cells, effecting osteoblast differentiation.^42^ The presence of BMP-2, BMP-4 or BMP-7 will also be contributing factors. Indeed, reparative responses have been reported for TGF-β1,^43^ BMP-7,^44^ BMP-2^45^ and FGF/IGF^46^ when examined in various in vivo or ex vivo wound repair models. There is increasing recognition in the literature that other ECM proteins play a role in stimulating mineralised tissue repair processes. The SLRPs decorin and biglycan can bind to growth factors and cytokines including TGF-β^14,15^ and BMP-2^16^ and have been proposed to play major roles in sequestering growth factors to the matrix, protecting them from proteolysis and modulating growth factor activity^14^ Biglycan has been proposed roles during early proliferative and osteogenic differentiation stages^17^ and can directly interact with cell surface receptors to enhance BMP-2-induced osteoblasts.^16^ Similarly, osteogenic signalling roles, leading to stimulation of reparative dentinogenesis using in vivo injury models, have been proposed for matrix proteins BSP and MEPE.^47^ DDM matrix may, therefore, provide enhanced osteogenic potency not only via synergistic action of combinations of growth factors, at physiological levels, but also by the contributory interaction with other ECM proteins.

The application of DDM for regenerative medicine would require its incorporation into a scaffold into which cells would be able to migrate efficiently. In an attempt to mimic this situation, the migration of the DPSCs into collagen gels containing 1 or 10 µg/mL DDM was assessed. This scenario could be considered to mimic the in vivo migration of DPSCs through a collagenous pulpal matrix infused with DDM. The presence of DDM did not appear to adversely affect the behaviour of the DPSCs, and they were able to penetrate to an approximate depth of 50 µm into the gel. The migratory process would have required degradation of the collagen matrix which could be facilitated by metaloproteinases released by the DPSC during migration, although a proteolytic contribution from MMPs reported as present in DDM^19^ is also possible. Cell numbers were higher after 72 h in culture, which could partially be attributable to maintenance of cell proliferative activity during culture. Within this experimental set-up, we could not confirm chemotactic properties of DDM for MSCs, which could be expected due to the previous purported presence of factors such as TGF-β1, PDGF and IGF-I and IL-1, IL-6 and tumour necrosis factor-alpha (TNF-α).^12,19^ This may be due to the higher concentrations of DDM employed in this study as cells often first sense chemotactic gradients at much lower levels at sites within the tissue remote to the release of the stimuli. The results, however, clearly indicate that the presence of DDM appeared to have no negative effect towards cell migration, repelling cells, thus supporting its potential for regenerative therapy applications.

In summary, the DDM components are able to stimulate cell expansion, promote cell survival through reduced apoptosis and promote osteogenic or odontogenic differentiation. Significantly, the results presented herein indicate that bioactive proteins in DDM facilitate signalling at physiological concentrations in stimulating these sequential wound healing events associated with mineralised tissue repair. Enhanced effects on cell proliferation and cell survival were abolished when the DDM was degraded with proteases and collagenase, indicating that bioactivity is achieved through intact proteins extractable from dentine. The study provides further support for a potential for the incorporation of DDM into scaffolds to stimulate dentine bridge formation below dental restorations. Close parallels with osteogenesis would imply application of the knowledge herein to the wider field of bone regeneration, where the major clinical challenges are accelerating healing and restoring effective signalling efficiency where bone repair is compromised.
